# *Aegilops crassa* Cytotypes in Some Regions of Türkiye

**DOI:** 10.3390/plants13213096

**Published:** 2024-11-03

**Authors:** Solmaz Najafi

**Affiliations:** Department of Field Crops, Faculty of Agriculture, Van Yuzuncu Yil University, 65090 Van, Türkiye; solmaznajafi@yyu.edu.tr

**Keywords:** *Aegilops crassa*, cytotype, tetraploid, hexaploid, DNA content, flow cytometry

## Abstract

A new hexaploid cytotype of *Aegilops crassa* has been identified in Türkiye. To assess the ploidy levels of native populations, 50 samples from Adıyaman, Batman, Bitlis, Diyarbakır, Hakkari, Mardin, Siirt, Şanlıurfa, Şırnak, and Van were analyzed using flow cytometry and cytogenetic techniques. DNA content was determined by comparison with standard plants. Results confirmed two cytotypes in Türkiye: tetraploid populations from Batman, Bitlis, Diyarbakır, Hakkari, Mardin, Siirt, Şanlıurfa, and Şırnak, and hexaploid accessions from Adıyaman and Van. Ten metaphase plates were analyzed. The tetraploid cytotype exhibited chromosome lengths of 8.95 ± 0.27 to 13.96 ± 0.13 µm, a total genome length of 165.51 ± 0.34 µm, and nuclear DNA content of 18.53 ± 0.29 to 20.37 ± 0.49 pg. Most chromosomes were metacentric, except for chromosomes 7, 8, 10, and 12, which were submetacentric. Two satellite pairs were found on chromosomes 4 and 10. The hexaploid cytotype showed chromosome lengths of 8.90 ± 0.16 to 14.06 ± 0.06 µm, a total genome length of 230.47 ± 0.23 µm, and nuclear DNA content of 33.40 ± 0.52 to 35.01 ± 0.31 pg. Most chromosomes were also metacentric, with three satellite pairs on chromosomes 3, 6, and 10. In conclusion, both tetraploid (2n = 2x = 28) and hexaploid (2n = 6x = 42) cytotypes of *Ae. crassa* exist in Türkiye, with the hexaploid cytotype having potential for wheat breeding programs.

## 1. Introduction

The genus *Aegilops*, commonly referred to as “goat grass” [1], encompasses numerous grass species with significant historical and botanical relevance. *Aegilops* was notably mentioned in Theophrastus’ botanical treatise Enquiry into Plants, a pivotal reference for botanical knowledge in antiquity and the Middle Ages [2]. The name *Aegilops* is derived from the Greek term “aegilos”, which may translate as “a herb cherished by goats” or “a herb resembling a goat”, in reference to the characteristic whiskery-awned spikelets of certain species [3,4]. Taxonomic classifications of *Aegilops* have evolved significantly since Linnaeus [5], with various taxonomists offering distinct perspectives. Zhukovsky [6] described 20 species, organizing them into nine sections, while Eig [7] recognized 22 species, distributed across two subgenera and six sections. Subspecies-level classifications have further diverged, with van Slageren [8] presenting a distinct perspective. Hammer [9] proposed a classification system that distinguishes taxa based on differences in chromosome numbers, morphology, or geographical distribution, recognizing them as separate subspecies.

Species within the *Aegilops* genus form an allopolyploid series, encompassing diploids (2n = 2x = 14), allotetraploids (2n = 4x = 28), and allohexaploids (2n = 6x = 42) [10]. *Aegilops crassa* (*Ae. crassa*), one of the species within this genus, commonly known as Persian goat grass, is the focus of the current research. It is an annual, robust plant that typically reaches heights of 20–40 cm (excluding spikes). Morphologically, *Ae. crassa* shares a close resemblance with *Ae. tauschii*, though it is highly variable across diagnostic traits such as spikelet structure and awn development. This variability prompted Eig [7] to classify *Ae. crassa* into two varieties: var. typica and var. palaestina (now considered *Ae. vavilovii* Zhuk). Hammer [9] later subdivided *Ae. crassa* into subspecies and forms, further highlighting its morphological diversity. As a steppic element, *Ae. crassa* is predominantly distributed across semi-desert regions of western Asia, extending into Transcaucasia, Turkmenistan, Uzbekistan, and other regions [11,12,13]. Recent studies, including those utilizing SSR, ISSR, and nuclear microsatellite markers, have documented extensive variation within *Ae. crassa* populations in Iran [14,15,16,17]. The species thrives in diverse habitats, including degraded steppe, forest edges, and disturbed sites, and it demonstrates significant drought tolerance, flourishing in areas with annual rainfall between 150 and 350 mm. Two cytotypes of *Ae. crassa* have been identified: an allotetraploid (2n = 4x = 28; DcDcXcXc) and an auto-allohexaploid (2n = 6x = 42; DcDcXcXcDD), both of which display morphological similarities [2].

Although *Ae. crassa* is widely distributed throughout Türkiye, no efforts have been made to differentiate between these two cytotypes in 90% of the country’s regions. Furthermore, due to the morphological similarities between these two cytotypes, many botanists are uncertain about their separation. Therefore, it is essential to conduct flow cytometry and karyological analyses to confirm this differentiation. As a result, there exists a significant research gap regarding the identification and classification of Aegilops cytotypes across various regions of Türkiye. To address this gap, the present study aims to investigate and identify these cytotypes through comprehensive chromosomal and karyotypic analysis, focusing on the nuclear genome characteristics of tetraploid and hexaploid cytotypes. Additionally, this research seeks to identify chromosomal markers and satellite chromosomes that distinguish these cytotypes, thereby contributing valuable insights into the genetic diversity and chromosomal structure of this species in Türkiye.

## 2. Results

### 2.1. Determination of Nuclear DNA Content

**Figure 1:**
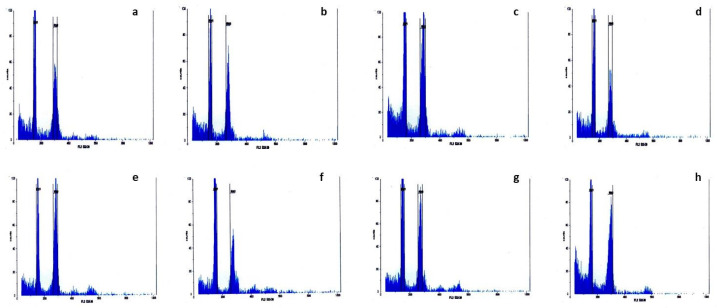
Flow cytometry histograms of relative fluorescence intensity were obtained from the analysis of DAPI-stained chromosome suspensions prepared from the standard plant (Hordeum vulgare) and tetraploid *Ae. crassa* samples. (**a**) Batman, (**b**) Bitlis, (**c**) Diyarbakır, (**d**) Hakkari, (**e**) Mardin, (**f**) Siirt, (**g**) Şanlıurfa, and (**h**) Şırnak.

**Figure 2:**
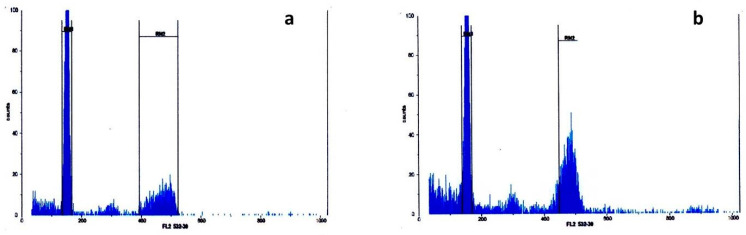
Flow cytometry histograms of relative fluorescence intensity; obtained from the analysis of DAPI-stained chromosome suspensions prepared from the standard plant (Hordeum vulgare) and hexaploid *Ae. crassa* samples. (**a**) Adiyaman, (**b**) Van.

**Figure 3:**
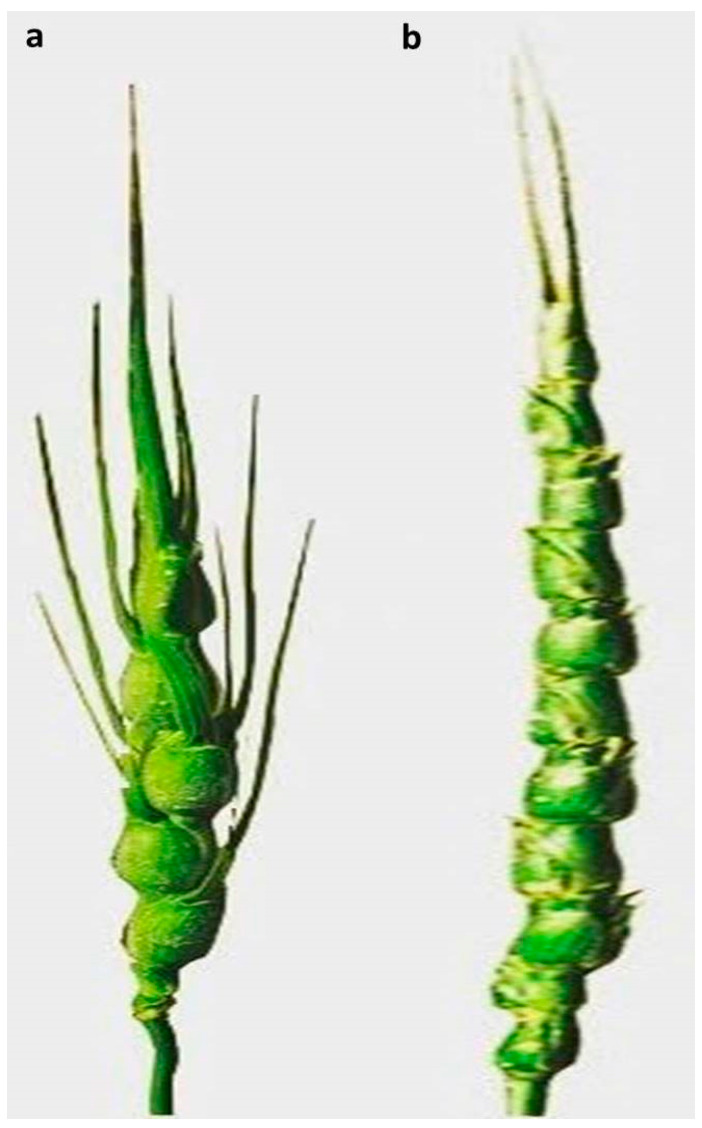
The spike of *Ae. crassa*; (**a**): *Ae. crassa* (4x); (**b**): *Ae. crassa* (6x).

By simultaneously analyzing the control (standard) plant and the test samples, the fluorescence intensity and G1 peak of each *Ae. crassa* sample were compared to those of the standard (Figure 1 and Figure 2). The absolute nuclear DNA content of each sample, expressed in picograms (pg), was calculated using the average fluorescence intensity values of the G1 peaks from both the standard and the sample. These values were compiled into a corresponding table (Table 1). The nuclear DNA content analysis of *Ae. crassa* accessions collected from different regions of Türkiye revealed two distinct ploidy levels, tetraploid and hexaploid, among the 50 populations sampled from ten regions. All populations from Batman, Bitlis, Diyarbakır, Hakkari, Mardin, Siirt, Şanlıurfa, and Şırnak were tetraploid, while all accessions from Adıyaman and Van were hexaploid (Table 1; Figure 1 and Figure 2). The spike morphology of both tetraploid and hexaploid cytotypes, which shows the differences between the two ploidy levels, is indicated in Figure 3.

Table 1 presents the mean nuclear DNA content (MDC) of *Ae. crassa* across various accessions collected from different regions of Türkiye. The values are expressed in picograms per two haploid C (pg/2C) and include the corresponding standard errors (SE) to provide insight into the variability of DNA content within each accession. The data indicate notable differences in mean DNA content between the tetraploid and hexaploid accessions. Specifically, the accession from Van exhibits the highest nuclear DNA content at 34.05 ± 0.31 pg/2C, while the accessions from Şırnak demonstrate the lowest DNA contents, measuring 18.55 ± 0.28 pg/2C.

### 2.2. Karyo-Morphometric Analyses

**Figure 4:**
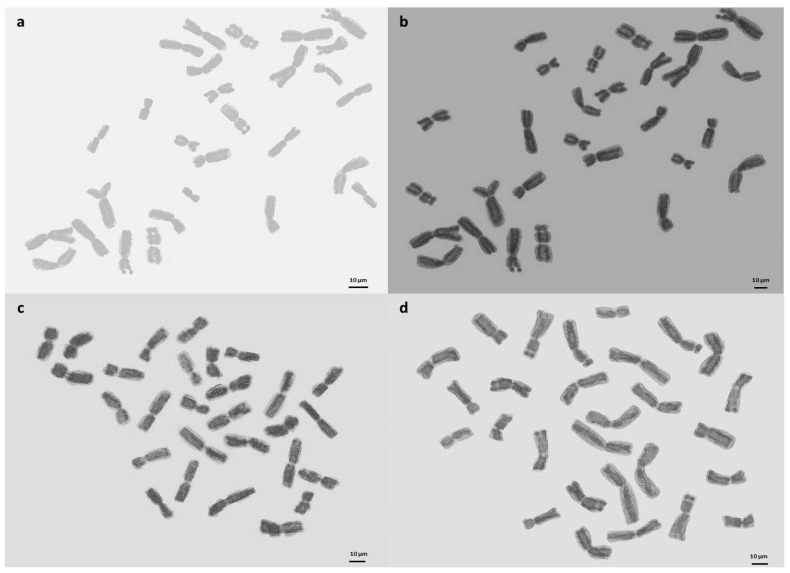
Micrographies of tetraploid metaphase plates (2n = 4x = 28) of *Ae. crassa* cytotype 1. (**a**) Batman; (**b**) Bitlis; (**c**) Diyarbakir; (**d**) Hakkari. Scale bar = 10 μm.

**Figure 5:**
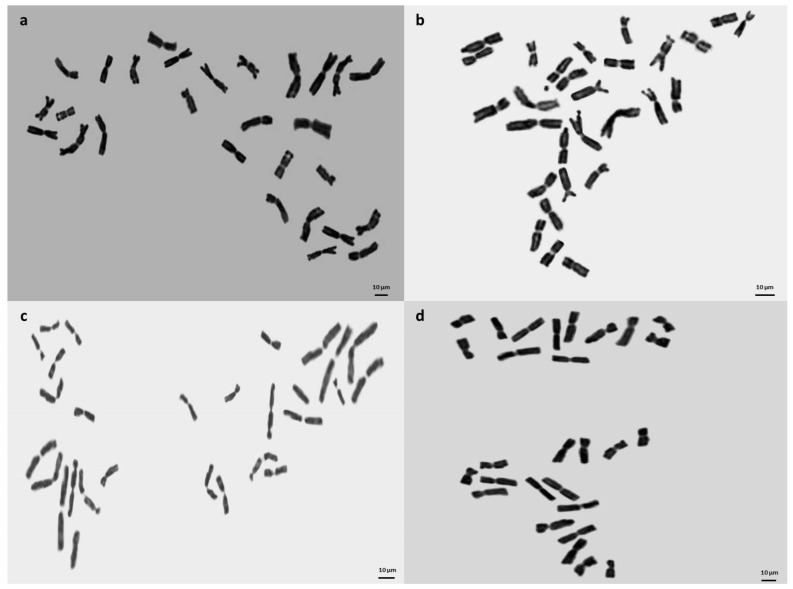
Micrographies of tetraploid metaphase plates (2n = 4x = 28) of *Ae. crassa* cytotype 1. (**a**) Mardin; (**b**) Siirt; (**c**) Şanliurfa; (**d**) Şirnak. Scale bar = 10 μm.

**Figure 6:**
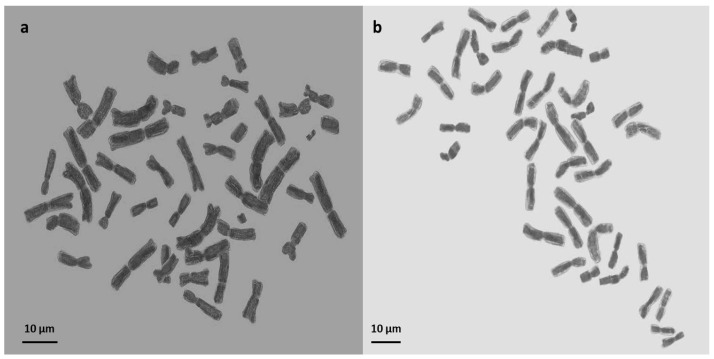
Micrographies of hexaploid metaphase plates (2n = 6x = 42) of *Ae. crassa* cytotype 2. (**a**) Adiyaman; (**b**) Van. Scale bar =10 μm.

**Figure 7:**
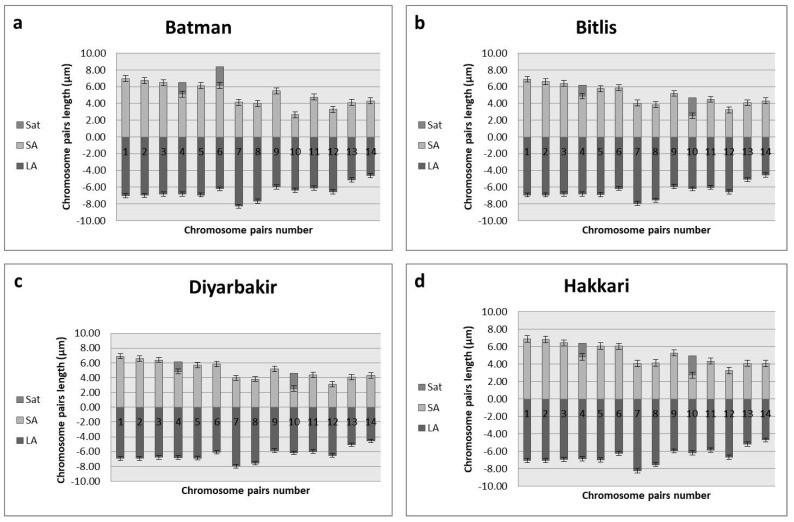
Haploid ideogram of tetraploid cytotypes of *Ae. crassa* (2n = 4x = 28); (**a**) Batman; (**b**) Bitlis; (**c**) Diyarbakir; (**d**) Hakkari.

**Figure 8:**
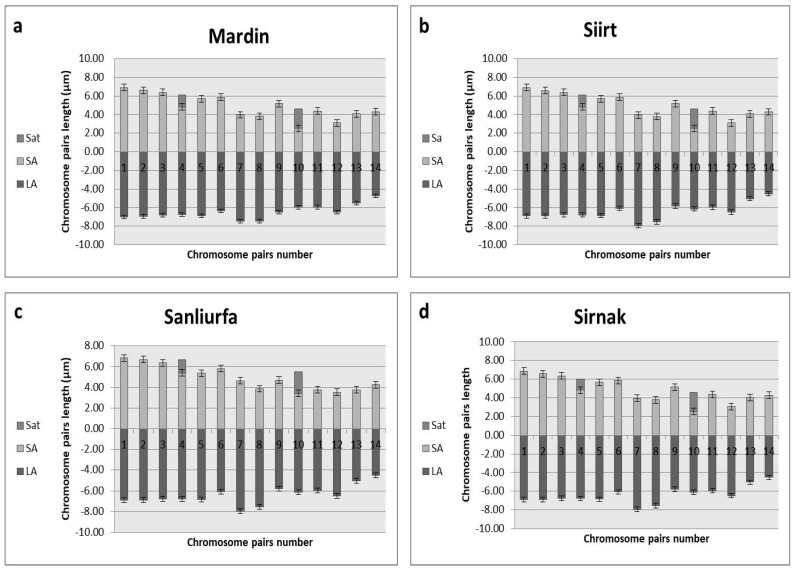
Haploid ideogram of tetraploid cytotypes of *Ae. crassa* (2n = 4x = 28); (**a**) Mardin; (**b**) Siirt; (**c**) Şanliurfa; (**d**) Şirnak.

**Figure 9:**
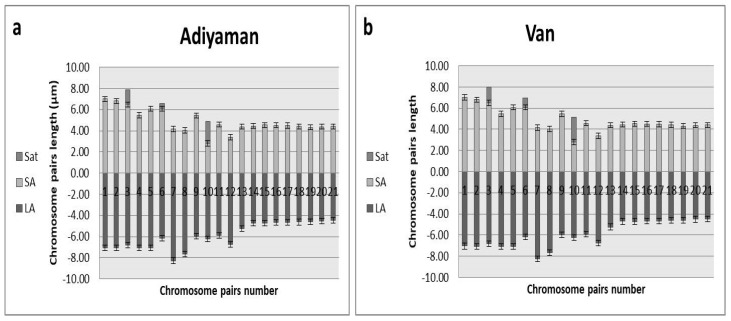
Haploid ideogram of hexaploid cytotypes of *Ae. crassa* (2n = 6x = 42); (**a**) Adiyaman; (**b**) Van.

To validate the ploidy levels determined by flow cytometry, cytological studies were conducted on both tetraploid and hexaploid samples. These analyses corroborated the flow cytometry findings, confirming that the indigenous Turkish *Ae. crassa* samples exhibit two distinct ploidy levels: tetraploid and hexaploid. Micrographies of tetraploid and hexaploid metaphase plates for each cytotype were constructed using five metaphase cells from each sample, with one representative metaphase plate selected and illustrated for each region (Figure 4, Figure 5 and Figure 6). The karyo-morphometric analyses of all spread chromosomes from the optimal metaphase plates revealed the two distinct ploidy levels for *Ae. crassa*: 2n = 4x = 28 (tetraploid) and 2n = 6x = 42 (hexaploid) (Table 2 and Table 3). These analyses facilitated the identification of eight tetraploid and two hexaploid cytotypes across the different regions. The haploid idiograms of the two cytotypes were established from the mean measurements obtained from the somatic metaphase chromosomes of the identified cytotypes across ten different accessions (Figure 7, Figure 8 and Figure 9).

In the chromosomal analysis of the two cytotypes, the tetraploid cytotype exhibited a longest chromosome measuring 13.96 ± 0.13 μm, while the shortest chromosome measured 8.95 ± 0.27 μm; both chromosomes were classified as metacentric. In contrast, for the hexaploid cytotype, the longest chromosome measured 14.06 ± 0.06 μm, and the shortest measured 8.90 ± 0.16 μm, with both also being metacentric. 

### 2.3. Cytotype and Idiogram Characters

#### 2.3.1. Cytotype 1: *Ae. crassa* (2n = 4x = 28), Tetraploid

Cytotype 1 was identified in populations sampled from the southeastern regions of Türkiye. This cytotype is characterized by relatively large chromosomes, ranging in length from 9.87 μm to 13.96 μm, with a total mean chromosome length of 165.51 μm. The karyotype consists of nine pairs of metacentric chromosomes, three pairs of submetacentric chromosomes, one pair of metacentric chromosomes with satellites, and one pair of submetacentric chromosomes with satellites.

#### 2.3.2. Cytotype 2: *Ae. crassa* (2n = 6x = 42), Hexaploid

Cytotype 2 was identified in populations sampled from a province in the southern region of Türkiye (Adıyaman) and in the easternmost province (Van). The chromosomes range in length from 8.96 μm to 14.06 μm, with a mean total length of 230.47 μm. The karyotype formula comprises fifteen pairs of metacentric chromosomes, three pairs of submetacentric chromosomes, two pairs of metacentric chromosomes with satellites, and one pair of submetacentric chromosomes with satellites.

### 2.4. Comparision of DNA Content and Karyological Parameters in Two Cytotypes of Ae. crassa

The results indicated that the tetraploid genotypes of *Ae. crassa* collected from different regions in Türkiye exhibited significant differences in DNA content (*p* < 0.01) (Table 4). However, the karyological parameters did not show significant variations among the regions.

The comparison of average DNA content across different regions (Table 5) revealed that the highest DNA content was found in the tetraploid genotypes from the Batman region, with a mean value of 20.49 ± 0.28 pg, categorizing it in a separate statistical group. Conversely, the lowest DNA content was recorded in the genotypes collected from the Şırnak region.

The results indicated that the hexaploid genotypes of *Ae. crassa* collected from various regions in Türkiye do not exhibit significant differences in DNA content or other karyological parameters at *p* < 0.01 (Table 6).

The comparison of average DNA content across different regions (Table 7) revealed that the highest DNA content was observed in the hexaploid genotypes from the Van region, with a mean value of 35.05 ± 0.18 pg. Conversely, the lowest DNA content was recorded in the genotypes collected from the Adıyaman region.

## 3. Discussion

Chromosomal findings are used in two primary ways for the classification and differentiation of cytotypes. The first method is descriptive, where chromosomal characteristics are compared to other morphological traits, such as the number of chromosomes being analogous to the number of stamens. This includes considerations of chromosome shape and type alongside phenotypic traits like leaf and petal shapes or the presence of various phenolic compounds. The second method provides specific insights from chromosome number and homology, which are crucial for understanding pairing behavior during meiosis. Mating behavior influences the reproductive success of hybrids, thereby shaping the reproductive strategies and diversity patterns within populations. Both aspects are essential for interpreting chromosomal data, with the analytical aspect being more significant in systematic biological (phylogenetic) studies, while the descriptive aspect holds greater importance for taxonomic (phenetic) purposes. Given that species are typically defined by their chromosomal number, this trait is a valuable taxonomic characteristic. Within a species, distinguishing different cytotypes involves examining chromosome morphology through classical cytogenetics, focusing on features such as chromosome length, type, centromere position, satellite presence, and the analysis of the plant’s morphological traits. Together, these methods provide a reliable diagnostic approach for identifying cytotypes [18,19,20,21]. According to these issues, a review of the opinions and findings of prominent scientists in plant taxonomy and biosystematics is conducted first, followed by a comparison of the findings of the present study with those of other researchers. Additionally, due to the significant morphological similarities between the tetraploid and hexaploid cytotypes in *Ae. crassa*, many researchers who have investigated this topic utilize flow cytometry and karyological analyses alongside apparent morphological findings to confirm this critical distinction.

The diversity within *Aegilops* is profound, characterized by a complex allopolyploid series of diploid (2n = 2x = 14), allotetraploid (2n = 4x = 28), and allohexaploid (2n = 6x = 42) species [10]. This polyploidy highlights the genomic complexity and evolutionary dynamics within the genus. Nuclear genome sizes vary significantly among *Aegilops* species, with diploids ranging from 4.84 to 7.52 pg, tetraploids from 9.59 to 12.64 pg, and hexaploids from 16.22 to 17.13 pg [11,12]. These findings emphasize the genetic richness and adaptability present in *Aegilops* species. Chromosome morphology within *Aegilops* is predominantly symmetric, with centromeres located in median or submedian positions. However, certain species, such as *Ae. caudata, Ae. umbellulata, Ae. comosa,* and *Ae. uniaristata*, along with their derived allopolyploids, exhibit asymmetric karyotypes [18,22]. *Ae. crassa* exists in two cytotypes: an allotetraploid (2n = 4x = 28, genome DcDcXcXc) and an auto-allohexaploid (2n = 6x = 42, genome DcDcXcXcDD) [2]. While both cytotypes share morphological similarities, the tetraploid form exhibits more robust, moniliform spikes, whereas the hexaploid form presents more cylindrical spikes [23]. Geographically, the tetraploid cytotype is more widespread, whereas the hexaploid cytotype is restricted to northern Afghanistan and northeastern Iran, reflecting their different evolutionary timelines, representative of an ancient allotetraploid and a more recent hexaploid derivative [24,25]. The origin of the allotetraploid *Ae. crassa* is attributed to hybridization between two diploid species, while the hexaploid likely emerged from a hybridization event between the tetraploid *Ae. crassa* and *Ae. tauschii*. F1 hybrids from these crosses were initially sterile but regained fertility following chromosome doubling, resulting in fertile polyploids. This genomic composition was verified through cytogenetic studies showing the presence of seven ring bivalents in F1 hybrids between tetraploid *Ae. crassa* and *Ae. tauschii* [26,27].

The first chromosome count for the tetraploid *Ae. crassa* was reported by Emme [28], although no mention was made of satellite chromosomes. Later, Chennaveeraiah [18] conducted a more detailed karyotypic analysis, identifying a symmetrical karyotype with two pairs of satellite chromosomes: one pair with a median centromere, and another with a submedian centromere. In contrast, for the hexaploid cytotype, three pairs of satellite chromosomes were observed, with both cytotypes sharing submedian chromosomes and lacking subterminal ones [2]. Species within the *Aegilops* genus are invaluable to wheat breeding programs, as they contribute critical traits such as pest resistance, tolerance to abiotic and biotic stress, and enhanced grain quality [29,30,31,32,33,34,35]. The identification and reporting of *Aegilops* cytotypes from various regions are crucial for advancing research in plant breeding, taxonomy, systematics, and biodiversity. While the allotetraploid cytotype of *Ae. crassa* is broadly distributed, the hexaploid cytotype is geographically restricted to specific areas in northern Afghanistan and northeastern Iran. The occurrence of mixed populations in these regions suggests that the hexaploid cytotype likely originated there.

Türkiye ranks first globally in terms of the wild wheat species it hosts, with all the relatives that contribute to modern wheat and that formed the first gene pool found within the country. The presence of both tetraploid and hexaploid cytotypes of *Ae. crassa* in Türkiye was first documented by Badaev *et al.* in 1998. Tetraploid cytotype samples were recorded in the Urfa region, while hexaploid cytotypes were found in the Menemen region of Izmir [36]. Subsequent reports have confirmed the presence of both *Ae. crassa* cytotypes in other regions, including Adıyaman, Ankara, and Siirt (tetraploid cytotype), as well as Kırıkkale and Tufanbeyli (hexaploid cytotype) [37]. Studies have also identified *Aegilops* species across three ploidy levels, namely, diploid, tetraploid, and hexaploidy, from various regions of Türkiye. However, in this particular research, only the hexaploid cytotype (6x = 42) of *Ae. crassa* has been documented [38,39,40].

In the current study, the nuclear DNA content of the tetraploid cytotype ranged from 18.53 to 20.37 pg, consistent with previous findings by Eilam et al. [11,12], who reported a value of 21.72 pg, as well as Najafi et al. (2022) [41], who measured 20.08 pg. In contrast, the nuclear DNA content of the hexaploid cytotype ranged from 33.40 to 35.01 pg. These results are comparable to the findings of Kimber and Tsunewaki [42] (33.63 pg) and Najafi et al. (2022) [41] (31.59 pg), indicating a similar degree of variation in nuclear DNA content between different studies and geographic populations. In terms of chromosomal structure, our study found that chromosomes in the tetraploid cytotype were predominantly metacentric, except for chromosomes 7, 8, 10, and 12, which were submetacentric. Two pairs of satellite chromosomes were observed on the short arms of chromosomes 4 and 10, with average sizes of 1.38 µm and 2.22 µm, respectively. These findings contrast with those of Ranjbar et al. [14], who reported no satellite chromosomes in their tetraploid cytotypes, as well as Emme [28], who similarly did not mention the presence of satellite chromosomes in tetraploid cytotypes.

For the hexaploid cytotype, the chromosome lengths in our study ranged from 8.90 ± 0.16 to 14.06 ± 0.06 µm, with a total genome length of 230.47 µm. These results are comparable to those of Ranjbar et al. [14], who reported chromosome lengths ranging from 7.53 ± 0.34 to 12.95 ± 0.56 µm and a total genome length of 217.39 µm. The chromosomal structure of the hexaploid cytotype was mainly metacentric, but chromosomes 7, 8, 10, and 12 were submetacentric. Additionally, three pairs of satellite chromosomes were identified on the short arms of chromosomes 3, 6, and 10, with average sizes of 1.39 µm, 0.53 µm, and 2.11 µm, respectively. These findings are consistent with those reported by Ranjbar et al. [14], supporting the similarity of hexaploid cytotypes across studies.

## 4. Materials and Methods

### 4.1. Plant Materials

In this study, 50 accessions of *Ae. crassa* were collected from diverse regions of Türkiye during the 2020–2021 growing seasons (Table 8). The research was conducted at two key facilities: nuclear DNA content was measured using flow cytometry (PARTEC, CyFlow Space, Nürnberg, Germany) at the Plant Genetics and Cytogenetics Laboratory, Faculty of Agriculture, Namık Kemal University, Türkiye. Chromosome analysis was carried out at the Cytogenetics Laboratory, Faculty of Agriculture, Van Yüzüncü Yıl University, Türkiye. The accessions were cultivated under controlled greenhouse conditions for morphological characterization. DNA extraction was performed to estimate nuclear DNA content via flow cytometry, and karyotypic analysis was conducted to evaluate chromosomal characteristics.

### 4.2. Ploidy Determination Using Flow Cytometry and Nuclear DNA Content Estimation

Nuclear DNA content was determined using flow cytometry following standard protocols [43,44,45]. Fresh leaf tissues from *Ae. crassa* (test material) and barley (Hordeum vulgare, 2n = 2x = 14) were utilized as the internal standard, with a known nuclear DNA content of 10.68 pg. The leaf samples were placed between damp filter papers in petri dishes and stored under laboratory conditions prior to analysis. Nuclear DNA content was measured using propidium iodide (PI) staining, following the manufacturer’s protocol. Briefly, 20 mg of fresh green leaf tissue from both the test and standard plants were chopped on ice with 500 μL of extraction buffer and crushed with a scalpel for 30–60 s. The resulting homogenate was gently shaken for 10–15 s, filtered through 50 µm filters (Celltrics, PARTEC, Nürnberg, Germany), and incubated in the dark with 2 mL of DAPI staining solution for 30–60 min. The samples were then analyzed using a flow cytometer, which produced a distinct G1 peak. The fluorescence intensity corresponding to the nuclei of the standard plant was established as a reference, and the G1 peak for each sample was recorded. Nuclear DNA content (in pg) was calculated based on the fluorescence intensity ratio between the G1 peaks of the sample and the standard, using the following equation:

$$NuclearDNA=\frac{Fluorescentintensityoftheplantsample}{Fluorescentintensityofstandardplantsamples}\times standardplantDNAcontent\left(pg\right)$$

To quantify the nuclear DNA content for each sample from the various regions, three replicates were performed. The mean value was then calculated based on measurements taken from five distinct locations within each region.

### 4.3. Karyo-Morphometric Analyses

For chromosome analysis, root tip meristems were pretreated with 8-hydroxyquinoline solution and subsequently fixed in Lewitsky solution. After pretreatment, the root tips were rinsed with distilled water and hydrolyzed in 1N HCl for 10 min at 60 °C. The root tips were then stained with aceto-orcein. For each accession, ten metaphase plates were prepared, and chromosome counting was performed using the squash method. Various chromosomal parameters were measured, including somatic chromosome number, total chromosome length (TCL), long arm length (LA), short arm length (SA), satellite length (Sat), arm ratio (AR), centromeric index, chromosome types, and karyotype formula. These measurements were carried out using MicroMeasure 3.3 software [46,47].

### 4.4. Statistical Analysis

Nuclear DNA content and chromosome analyses were performed with three replicates for each experiment. The study was designed following a completely randomized design (CRD). Data were subjected to one-way ANOVA, and mean values were compared using Duncan’s multiple range test at a significance level of *p* < 0.01. All statistical analyses were conducted using StatGraphics version XVII.

## 5. Conclusions

This study provides significant insights into the nuclear DNA content, chromosomal structure, and distribution of *Ae. crassa* cytotypes, particularly in Türkiye. Our findings emphasize the genetic diversity and complexity of both tetraploid and hexaploid cytotypes, which have important implications for taxonomy, plant breeding, and the conservation of genetic resources. Notably, the identification of satellite chromosomes in the tetraploid cytotype, previously unreported, offers a new perspective on the chromosomal architecture of *Aegilops*. The observed genetic variability and geographic distribution of hexaploid cytotypes underscore the evolutionary potential of *Aegilops* species in enhancing stress tolerance and resistance in wheat breeding programs. Further research into the genomic mechanisms underlying these variations will be crucial for advancing our understanding of polyploid evolution and for leveraging *Aegilops* genetic resources in crop improvement strategies. Moreover, detailed data on the distribution of tetraploid and hexaploid cytotypes across different regions of Türkiye remains limited. Continued investigation in this area is essential and encourages researchers to explore further.

## Figures and Tables

**Table 1:** The mean nuclear DNA content of *Ae. crassa* in accessions of Türkiye.

Accessions	2n	MDC (pg/2C) ± SE	Accessions	2n	MDC (pg/2C) ± SE	Accessions	2n	MDC (pg/2C) ± SE
Adıyaman	42	33.41 ± 0.52	Hakkari	28	20.33 ± 0.30	Şırnak	28	18.55 ± 0.28
Batman	28	20.49 ± 0.48	Mardin	28	19.72 ± 0.27	Van	42	34.05 ± 0.31
Bitlis	28	20.13 ± 0.08	Siirt	28	19.49 ± 0.40			
Diyarbakır	28	19.91 ± 0.18	Şanlıurfa	28	18.67 ± 0.53			

MDC: mean DNA content; SE: standard error; pg: picogram.

**Table 2:** Karyo-morphometric parameters in tetraploid accessions of *Ae. crassa*.

Chr. No	TCL ± SE (µm)	LA ± SE (µm)	SA ± SE (µm)	AR ± SE (µm)	Sat	CI (SA/LA +SA) × 100 + SE	Chr. Type	KF
1	13.96 ± 0.13	7.00 ± 0.05	6.96 ± 0.09	1.01 ± 0.03	-	49.84 ± 0.15	m	18m + 6sm + 2m^sat^ + 2sm^sat^
2	13.72 ± 0.12	6.98 ± 0.11	6.74 ± 0.08	1.04 ± 0.01	-	49.10 ± 0.49	m	
3	13.30 ± 0.59	6.82 ± 0.18	6.49 ± 0.43	1.05 ± 0.05	-	48.73 ± 1.22	m	
4	13.31 ± 0.21	6.81 ± 0.17	5.12 ± 0.06	1.33 ± 02	1.38	38.49 ± 0.69	m	
5	13.07 ± 0.31	6.93 ± 0.28	6.14 ± 0.16	1.13 ± 0.02	-	47.01 ± 1.25	m	
6	12.36 ± 0.16	6.20 ± 0.09	6.16 ± 0.07	1.01 ± 06	-	49.87 ± 0.09	m	
7	12.43 ± 0.24	8.27 ± 0.05	4.16 ± 0.19	1.99 ± 0.04	-	33.45 ± 0.87	sm	
8	11.70 ± 0.33	7.67 ± 0.28	4.03 ± 0.05	1.90 ± 0.03	-	34.46 ± 0.52	sm	
9	11.50 ± 0.54	5.98 ± 0.09	5.52 ± 0.46	1.09 ± 0.07	-	47.95 ± 1.71	m	
10	11.26 ± 0.46	6.38 ± 0.35	2.66 ± 0.19	2.41 ± 0.05	2.22	23.70 ± 2.58	sm	
11	10.85 ± 0.73	6.09 ± 0.41	4.76 ± 0.31	1.28 ± 0.07	-	43.86 ± 0.13	m	
12	9.87 ± 0.31	6.56 ± 0.23	3.31 ± 0.09	1.98 ± 0.11	-	33.57 ± 0.34	sm	
13	9.23 ± 0.39	5.13 ± 0.11	4.11 ± 0.28	1.25 ± 0.09	-	44.44 ± 1.20	m	
14	8.95 ± 0.27	4.63 ± 0.11	4.31 ± 0.16	1.07 ± 0.02	-	48.21 ± 0.36	m	
Total	165.51 ± 0.34	6.53 ± 0.05	5.03 ± 0.02	1.40 ± 0.03	3.60	42.33 ± 0.83		

Chr. No: chromosome pairs number; TCL: total chromosome length; SE: standard error; LA: chromosome long arm; SA: chromosome short arm; AR: arm ratio; Sat: satellite; CI: centromeric index; KF: karyotype formula; m: metacentric; sm: submetacentric.

**Table 3:** Karyo-morphometric parameters in hexaploid accessions of *Ae. crassa*.

Chr. No	TCL	LA	SA	AR	CI (SA/LA + SA) ×100 + SE	SAT	Chr. Type	KF
1	14.06 ± 0.06	7.05 ± 0.04	7.01 ± 0.02	1.00 ± 0.01	49.88 ± 0.08	-	m	2n = 6x = 42 = 30m + 6sm + 4m^sat^ + 2sm^sat^
2	13.86 ± 0.23	7.06 ± 0.07	6.80 ± 0.17	1.04 ± 0.02	49.06 ± 0.42	-	m	
3	14.70 ± 0.58	6.81 ± 0.19	6.49 ± 0.42	1.05 ± 0.01	44.15 ± 1.31	1.39	m	
4	12.52 ± 0.99	7.06 ± 0.36	5.46 ± 0.65	1.30 ± 0.02	43.54 ± 1.74	-	m	
5	13.16 ± 0.38	7.09 ± 0.33	6.07 ± 0.05	1.17 ± 0.03	46.14 ± 1.00	-	m	
6	12.75 ± 0.44	6.15 ± 0.12	6.07 ± 0.18	1.01 ± 0.01	47.60 ± 0.50	0.53	m	
7	12.43 ± 0.24	8.27 ± 0.05	4.16 ± 0.19	1.99 ± 0.04	33.45 ± 0.87	-	sm	
8	11.70 ± 0.33	7.67 ± 0.28	4.03 ± 0.05	1.90 ± 0.03	34.46 ± 0.52	-	sm	
9	11.44 ± 0.44	5.99 ± 0.10	5.45 ± 0.35	1.10 ± 0.02	47.62 ± 1.19	-	m	
10	11.13 ± 0.25	6.23 ± 0.11	2.80 ± 0.05	2.23 ± 0.02	25.13 ± 0.27	2.11	sm	
11	10.50 ± 0.22	5.91 ± 0.15	4.58 ± 0.08	1.29 ± 0.05	43.67 ± 0.38	-	m	
12	10.16 ± 0.25	6.77 ± 0.21	3.39 ± 0.04	2.00 ± 0.04	33.37 ± 0.44	-	sm	
13	9.61 ± 0.30	5.23 ± 0.10	4.38 ± 0.20	1.20 ± 0.02	45.53 ± 0.71	-	m	
14	9.16 ± 0.12	4.71 ± 0.04	4.45 ± 0.09	1.06 ± 0.01	48.61 ± 0.37	-	m	
15	9.24 ± 0.21	4.71 ± 0.14	4.53 ± 0.03	1.04 ± 0.03	49.03 ± 0.11	-	m	
16	9.19 ± 0.24	4.68 ± 0.15	4.51 ± 0.01	1.04 ± 0.01	49.08 ± 1.4	-	m	
17	9.14 ± 0.14	4.65 ± 0.16	4.49 ± 0.05	1.04 ± 0.02	49.12 ± 1.21	-	m	
18	9.04 ± 12	4.63 ± 07	4.41 ± 0.06	1.05 ± 0.02	48.78 ± 0.41	-	m	
19	8.91 ± 17	4.59 ± 0.02	4.32 ± 0.07	1.06 ± 0.05	48.48 ± 0.22	-	m	
20	8.89 ± 21	4.51 ± 0.11	4.38 ± 0.02	1.03 ± 0.06	49.25 ± 0.03	-	m	
21	8.90 ± 16	4.49 ± 0.08	4.40 ± 0.11	1.02 ± 0.08	49.49 ± 0.20	-	m	
Total	230.47 ± 0.23	5.92 ± 0.03	4.87 ± 0.04	1.27	44.55 ± 0.48	4.03		

Chr. No: chromosome pairs number; TCL: total chromosome length; SE: standard error; LA: chromosome long arm; SA: chromosome short arm; AR: arm ratio; Sat: satellite; CI: centromeric index; KF: karyotype formula; m: metacentric; sm: submetacentric.

**Table 4:** Analysis of variance in DNA content and karyological parameters of tetraploid genotypes of *Ae. crassa* collected from different regions in Türkiye.

S.O.V	Df	Mean of Square					
		DNA Content	TCL	LA	SA	AR	CI
Region	7	1.58 **	13.84 ^ns^	0.01 ^ns^	0.02 ^ns^	0.0009 ^ns^	0.14 ^ns^
Error	16	0.12	45.96	0.03	0.09	0.007	1.19
Total	23						
F		12.92	0.30	0.38	0.21	0.13	0.12

TCL: total chromosome length; LA: chromosome long arm; SA: chromosome short arm; AR: arm ratio; CI: centromere index; **: significant at *p* < 0.01; ^ns^: not significant.

**Table 5:** The comparison of DNA content and karyological parameter averages in *Ae. crassa* tetraploid genotypes collected from different regions in Türkiye.

Region	DNA Content	TCL	LA	SA	AR	CI
Batman	20.49 ± 0.28 ^a^	165.51 ± 1.20 ^a^	6.53 ± 0.05 ^a^	5.03 ± 0.03 ^a^	1.39 ± 0.01 ^a^	42.33 ± 0.08 ^a^
Hakkari	20.34 ± 0.17 ^ab^	165.38 ± 1.18 ^a^	6.57 ± 0.07 ^a^	4.97 ± 0.07 ^a^	1.41 ± 0.03 ^a^	41.80 ± 0.60 ^a^
Bitlis	20.13 ± 0.05 ^ab^	161.99 ± 3.43 ^a^	6.45 ± 0.09 ^a^	4.87 ± 0.17 ^a^	1.43 ± 0.05 ^a^	41.81 ± 0.59 ^a^
Diyarbakir	19.91 ± 0.11 ^ab^	161.08 ± 4.30 ^a^	6.43 ± 0.10 ^a^	4.84 ± 0.20 ^a^	1.44 ± 0.06 ^a^	41.71 ± 0.69 ^a^
Mardin	19.72 ± 0.16 ^ab^	160.94 ± 4.43 ^a^	6.43 ± 0.11 ^a^	4.83 ± 0.21 ^a^	1.44 ± 0.06 ^a^	41.69 ± 0.70 ^a^
Siirt	19.49 ± 0.24 ^bc^	160.79 ± 4.57 ^a^	6.42 ± 0.11 ^a^	4.83 ± 0.21 ^a^	1.44 ± 0.06 ^a^	41.69 ± 0.70 ^a^
Sanliurfa	18.67 ± 0.31 ^cd^	160.58 ± 4.78 ^a^	6.41 ± 0.12 ^a^	4.82 ± 0.22 ^a^	1.44 ± 0.06 ^a^	41.70 ± 0.69 ^a^
Sirnak	18.55 ± 0.16 ^d^	160.24 ± 5.11 ^a^	6.40 ± 0.13 ^a^	4.81 ± 0.23 ^a^	1.44 ± 0.06 ^a^	41.70 ± 0.70 ^a^

TCL: total chromosome length; LA: chromosome long arm; SA: chromosome short arm; AR: arm ratio; CI: centromere index. Values with the same letters in each column are not statistically different at *p* < 0.01 based on Duncan’s multiple range test (DMRT).

**Table 6:** Analysis of variance on DNA content and karyological parameters of hexaploid genotypes of *Ae. crassa* collected from different regions in Türkiye.

S.O.V	df	Mean of Square					
		DNA Content	TCL	LA	SA	AR	CI
Region	1	0.60 ^ns^	4040.41 ^ns^	0.09 ^ns^	0.05 ^ns^	0.00009 ^ns^	0.14 ^ns^
Error	4	0.18	823.15	0.04	0.04	0.006	0.06
Total	5						
F		3.26	4.91	2.37	1.32	0.01	2.43

TCL: total chromosome length; LA: chromosome long arm; SA: chromosome short arm; AR: arm ratio; CI: centromere index; ^ns^: not significant.

**Table 7:** The comparison of DNA content and karyological parameter averages in *Ae. crassa* hexaploid genotypes collected from different regions in Türkiye.

Region	DNA Content	TCL	LA	SA	AR	CI
Adiyaman	34.42 ± 0.30 ^a^	188.28 ± 22.45 ^a^	5.92 ± 0.05 ^a^	4.87 ± 0.04 ^a^	1.34 ± 0.04 ^a^	44.55 ± 0.06 ^a^
Van	35.05 ± 0.18 ^a^	240.18 ± 6.69	6.17 ± 0.16 ^a^	5.04 ± 0.15 ^a^	1.34 ± 0.05 ^a^	44.24 ± 0.19 ^a^

TCL: total chromosome length; LA: chromosome long arm; SA: chromosome short arm; AR: arm ratio; CI: centromere index. Values with the same letters in each column are not statistically different at *p* < 0.01 based on Duncan’s multiple range test (DMRT).

**Table 8:** Geographical distribution of accessions.

Region	Location	Latitude	Longitude	Altitude
ADIYAMAN	1	37.8239	38.2623	966.2011
	2	37.7621	38.1534	736.7393
	3	37.7635	38.3279	718.2860
	4	37.8293	38.6978	723.6678
	5	37.8124	38.6126	707.6066
BATMAN	1	38.0373	41.2986	747.9836
	2	37.9634	41.2459	699.2628
	3	37.8254	41.2138	666.3896
	4	37.8299	41.3870	842.9821
	5	37.9671	41.1704	665.2117
BİTLİS	1	38.4308	42.0540	1954.2890
	2	38.5212	42.1943	1814.6393
	3	38.5087	41.9611	1976.5944
	4	38.7826	42.2836	1878.1182
	5	38.8209	42.6488	2037.6939
DİYARBAKIR	1	38.0020	39.9795	822.2459
	2	37.8743	40.6415	667.6980
	3	37.9559	40.2812	761.9961
	4	37.8989	40.0394	852.0756
	5	37.9111	40.4119	764.4158
HAKKARİ	1	37.6239	43.7402	2600.4914
	2	37.6481	44.0084	2573.5108
	3	37.6523	44.2537	2379.4649
	4	37.2783	44.3920	1916.4349
	5	37.3685	43.5230	1506.9818
MARDİN	1	37.3022	40.8184	784.7081
	2	37.2583	40.7325	598.9476
	3	37.2184	40.5560	533.5359
	4	37.2987	40.9240	1043.6800
	5	37.1949	40.7059	562.3227
SİİRT	1	37.9444	41.9436	883.3417
	2	37.9410	41.8861	794.8792
	3	37.9089	41.9026	880.4117
	4	37.9515	41.9979	1156.4062
	5	37.9346	42.0207	1133.9996
ŞANLIURFA	1	37.1521	38.6951	752.8925
	2	36.9794	38.9961	405.6635
	3	36.8344	39.0286	332.9841
	4	37.0007	39.1763	405.4978
	5	36.9870	38.5686	559.8538
ŞIRNAK	1	37.5201	42.4320	1199.5495
	2	37.5232	42.4485	1318.2267
	3	37.5113	42.4393	1118.5371
	4	37.5188	42.4521	1320.2921
	5	37.5113	42.4393	1118.5389
VAN	1	38.5303	43.3539	1706.7117
	2	38.5171	43.4002	1845.3378
	3	38.4132	43.2710	1832.9277
	4	38.3805	43.3157	1969.6559
	5	38.4436	43.5536	2422.8174

## Data Availability

The original contributions presented in this study are included in the article. Further inquiries can be directed to the corresponding author.
